# Multiplexed Microfluidic Platform for Parallel Bacterial Chemotaxis Assays

**DOI:** 10.21769/BioProtoc.5062

**Published:** 2024-09-05

**Authors:** Michael R. Stehnach, Richard J. Henshaw, Sheri A. Floge, Jeffrey S. Guasto

**Affiliations:** 1 Department of Mechanical Engineering, Tufts University, Medford, MA, USA; 2 Department of Physics, Brandeis University, Waltham, MA, USA; 3 Institute of Environmental Engineering, ETH Zürich, Zürich, Switzerland; 4 Department of Biology, Wake Forest University, Winston-Salem, NC, USA

**Keywords:** Microfluidics, Microfabrication, Chemotaxis, High-throughput screening, Bacteria, Serial dilution, Gradient generation, Photolithography, Soft lithography, Polydimethylsiloxane (PDMS)

## Abstract

The sensing of and response to ambient chemical gradients by microorganisms via chemotaxis regulates many microbial processes fundamental to ecosystem function, human health, and disease. Microfluidics has emerged as an indispensable tool for the study of microbial chemotaxis, enabling precise, robust, and reproducible control of spatiotemporal chemical conditions. Previous techniques include combining laminar flow patterning and stop-flow diffusion to produce quasi-steady chemical gradients to directly probe single-cell responses or loading micro-wells to entice and ensnare chemotactic bacteria in quasi-steady chemical conditions. Such microfluidic approaches exemplify a trade-off between high spatiotemporal resolution of cell behavior and high-throughput screening of concentration-specific chemotactic responses. However, both aspects are necessary to disentangle how a diverse range of chemical compounds and concentrations mediate microbial processes such as nutrient uptake, reproduction, and chemorepulsion from toxins. Here, we present a protocol for the multiplexed chemotaxis device (MCD), a parallelized microfluidic platform for efficient, high-throughput, and high-resolution chemotaxis screening of swimming microbes across a range of chemical concentrations. The first layer of the two-layer polydimethylsiloxane (PDMS) device comprises a serial dilution network designed to produce five logarithmically diluted chemostimulus concentrations plus a control from a single chemical solution input. Laminar flow in the second device layer brings a cell suspension and buffer solution into contact with the chemostimuli solutions in each of six separate chemotaxis assays, in which microbial responses are imaged simultaneously over time. The MCD is produced via standard photography and soft lithography techniques and provides robust, repeatable chemostimulus concentrations across each assay in the device. This microfluidic platform provides a chemotaxis assay that blends high-throughput screening approaches with single-cell resolution to achieve a more comprehensive understanding of chemotaxis-mediated microbial processes.

Key features

• Microchannel master molds are fabricated using photolithography techniques in a clean room with a mask aligner to fabricate multilevel feature heights.

• The microfluidic device is fabricated from PDMS using standard soft lithography replica molding from the master molds.

• The resulting microchannel requires a one-time calibration of the driving inlet pressures, after which devices from the same master molds have robust performance.

• The microfluidic platform is optimized and tested for measuring chemotaxis of swimming prokaryotes.

## Graphical overview


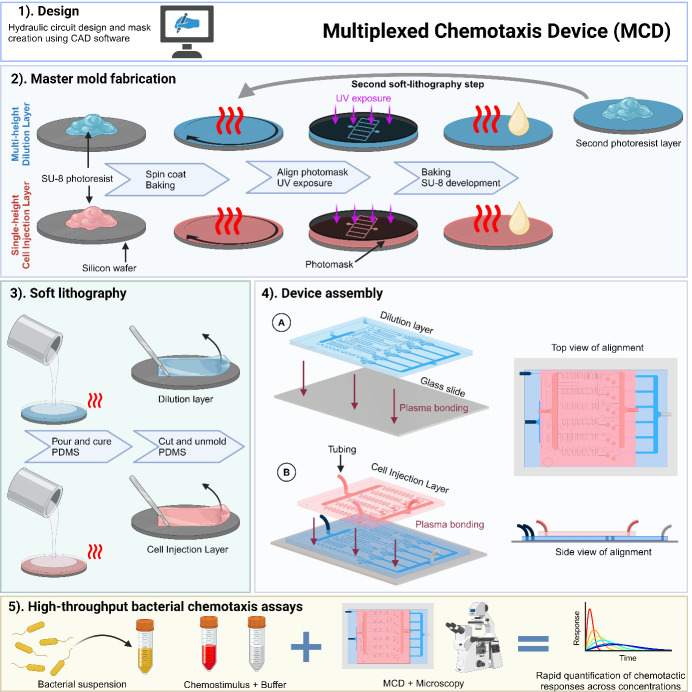


## Background

Many motile microorganisms use chemotaxis to navigate complex environments by detecting and responding to gradients in chemical stimuli [1–3]. Chemotaxis mediates many key biological processes including reproduction [4], microbial foraging [5], and infection [6]. Over the past two decades, microfluidics have been established as a crucial tool for studying chemotaxis [7,8] by enabling precise control of the chemical conditions experienced by swimming cells [9]. Applications have included modeling nutrient patches [5], precise and systematic variation of concentration gradients [10], drug dose–response quantification [11], and infectious disease diagnostics [12]. Microfluidic chemotaxis assays inherently rely on diffusion and device geometry to establish well-defined and reproducible chemical gradients. Passive devices [8,13,14] use relatively large reservoirs of chemoattractant loaded into micro-wells to produce long-lasting spatial gradients. Many gradient generators [15–17] stratify chemostimulant and buffer solutions via laminar flow patterning to establish steady gradients under continuous flow or slowly evolving gradients via diffusion in a stop-flow device. Porous membranes and hydrogel microfluidics produce steady-state gradients without subjecting microbes to an external flow [9,18]. Each of these methods produces tunable gradients to quantify aspects of microbial chemotaxis. However, there is a trade-off between high-throughput measurement of population-level cell responses to a diverse range of chemostimulants across a range of concentrations (e.g., micro-well techniques) versus high-spatiotemporal resolution of cell behavior within a defined single chemical gradient (e.g., gradient generators).

Here, we present the multiplexed chemotaxis device [19] (MCD). This high-throughput microfluidic platform capitalizes on the parallelization afforded by microfluidics [11,12,20] to conduct simultaneous microbial chemotaxis screening across gradient conditions spanning the microbe’s entire sensitivity range. The MCD comprises a two-layer architecture: (i) a serial dilution layer [11] produces five logarithmically diluted chemostimulant solutions (plus a control) and (ii) a cell injection layer introduces microbes and buffer. The MCD performs six stop-flow diffusion assays simultaneously on a single chip, where the performance has been fully characterized, validated, and benchmarked against conventional single gradient generator assays [19]. The serial dilution ratios are robust and customizable during the design stage. Compared to existing microfluidic techniques, the MCD has significantly higher throughput by enabling the simultaneous measurements of chemotactic responses across a range of concentration gradient conditions. This methodology has recently been applied to rapidly screen bacterial chemotaxis toward a wide range of identified metabolites produced by phage-infected cyanobacteria [21].

The MCD limitations are derived from its relative structural complexity. Whilst typical microfluidic devices consist of a single layer that can be fabricated in almost any microfabrication facility, the dilution layer of the MCD requires a mask aligner. Care must be taken with the alignment of the two PDMS layers, as a misalignment could render the device inaccurate or inoperable. Different choices of dilution ratios require a new dilution layer geometry fabricated to new design specifications. Finally, in the current mode of operation, each observation chamber is imaged once every few seconds due to limitations in automated microscopy. Whilst typically insufficient to capture bacteria motility dynamics, this can be mitigated by periodically capturing a short video of each individual assay at the cost of decreasing the sampling frequency across the MCD. Other potential applications of the MCD include but are not limited to accelerating microfluidic approaches to human health studies [22,23], chemical synthesis and drug discovery [24], and studying protists and antibiotic-resistant bacteria [25].

## Materials and reagents

**Reagents**

1. Bovine serum albumin (BSA) (Sigma-Aldrich, catalog number: A7906)
2. Fluorescein sodium salts (fluorescein) (Sigma-Aldrich, catalog number: F6377)
3. Polydimethylsiloxane mixture (PDMS) (SYLGARD^TM^ 184) (Sigma-Aldrich, catalog number: 761036)
4. SU-8 3050 photoresist (Kayaku Advanced Materials)
5. SU-8 2025 photoresist (Kayaku Advanced Materials)
6. Tridecafluoro-1,1,2,2-tetrahydrooctyl trichlorosilane (silanization agent) (Gelest Inc., catalog number: SIT8174.0)
7. Propylene glycol methyl ether acetate (photoresist developer) (Sigma-Aldrich, catalog number: 484431)
8. Ethanol (VWR, CAS number: 64-17-5)
9. Acetone (Sigma-Aldrich, CAS number: 67-64-1)
10. Isopropyl alcohol (Sigma-Aldrich, CAS number: 67-63-0)
11. Deionized (DI) water
12. Buffer solution cell resuspension/dilution. For example, a minimal motility medium or artificial seawater (ASW) as appropriate. The choice of media is organism-dependent; for example, the commonly used M9 minimal media for bacteria [26]. In the case of marine microbes, two common examples include artificial seawater amended with Provasoli’s ES solution (ESAW) [27,28] or the commercially available Instant Ocean^®^ sea salt

**Solutions**

1. Polydimethylsiloxane (PDMS) mixture (see Recipes)
2. BSA solution (see Recipes)
3. Fluorescein stock solution, 1 mM (see Recipe)
4. Fluorescein stock solution, 0.1 mM (see Recipes)

**Recipes**

1. **Polydimethylsiloxane (PDMS) mixture**
*Note: A 10:1 mass ratio of elastomer base to curing agent was used. The total mass of each component depends on the open volume on the mold.*
  **Table:** 

Reagent	Final concentration	Quantity or Volume
SYLGARD^TM^ 184 silicone elastomer base	90.9% wt	30 g
SYLGARD^TM^ 184 silicone elastomer curing agent	9.1% wt	3.0 g
Total (optional)	n/a	33 g
2. **BSA solution**
*Note: Filter mixture through a 0.2 μm syringe filter after mixing and store at 4 °C. Preferably use within two weeks.*
  **Table:** 

Reagent	Final concentration	Quantity or Volume
BSA	n/a	0.1 g
DI water	n/a	20 mL
Total (optional)	0.5% w.v.	20 ml
3. **Fluorescein stock solution (1 mM)**
  **Table:** 

Reagent	Final concentration	Quantity or Volume
Fluorescein sodium salts	n/a	0.0941 g
DI water	n/a	250 mL
Total (optional)	1 mM	250 mL
4. **Fluorescein stock solution (0.1 mM)**
  **Table:** 

Reagent	Final concentration	Quantity or Volume
1 mM fluorescein solution (Recipe 3)	n/a	25 mL
DI water	n/a	225 mL
Total (optional)	0.1 mM	250 mL

**Laboratory supplies**

1. Precision wipes (Kimtech Science, VWR, catalog number: 115-2029)
2. Silicon wafer (University Wafer, catalog number: 452)
3. Glass Petri dish (BRAND Petri dish) (Sigma-Aldrich, catalog number: BR455751)
4. 1.5 mm biopsy punch (Integra Miltex, VWR, catalog number: 95039-092)
5. Double-wide (75 mm × 50 mm) microscope slides (Corning, catalog number: 2947-75X50)
6. Tape (Scotch Magic Tape or Highland invisible tape, 6200)
7. Lab labeling tape (VWR, catalog number: VWRU89097-928)
8. 15 mL conical centrifuge tubes (Falcon, Fisher Scientific, catalog number: 352096)
9. 50 mL conical centrifuge tubes (Falcon, Fisher Scientific, catalog number: 352070)
10. 20 mL Luer lock plastic syringe (Codan, catalog number: 62.7602)
11. Tygon tubing (0.020” ID × 0.060” OD) (Masterflex, Fisher Scientific, catalog number: NC0578437)
12. 0.2 μm syringe filters (Filtropur, VWR catalog number: 103573-246)
13. Ratchet tubing clamp (Cole-Parmer Celcon, Fisher Scientific, catalog number: 11752703)
14. 23G Luer lock blunt syringe needles (CellLink, catalog number: NZ5231005001)
15. Retort stand (R&L Enterprises, Fisher Scientific, catalog number: 11779593)
16. Retort stand clamps (R&L Enterprises, Fisher Scientific, catalog number: 11517722)
17. Plastic Petri dishes (150 mm diameter) (VWR, catalog number: 25384-326)
18. Cutting knife (e.g., X-ACTO, catalog number: X3602)
19. Cutting pad (e.g., Fiskars, catalog number: 183720-1001)
20. Compressed nitrogen (e.g., Middlesex Gases & Technologies Inc., NI 3133)

## Equipment

1. Spin coater (Laurell Technologies Corporation, WS-400B06NPP-Lite Manual Spinner)
2. Wafer alignment tool (Laurell Technologies Corporation)
3. Mask aligner (OAI, model: 204IR Mask Aligner)
4. Stylus profilometer (Bruker Dektak)
5. Hot plate (Cimarec+^TM^, model: Stirring Hot Plate MA-1827)
6. Plasma oven (Plasma Etch Inc, model: PE-25)
7. Inverted microscope with motorized stage (Nikon, model: Ti-E)
8. Scientific camera (Andor, model: Zyla 5.5)
9. Pressure controller (Elveflow, model: OB1)
10. Vacuum desiccator (e.g., Nalgene, Thermo Fisher Scientific, catalog number: 5310-0250)
11. Mylar photomasks (Artnet Pro, formally CAD/Art Services)

## Software and datasets

1. AutoCAD from AutoDesk (https://www.autodesk.com/products/autocad). Free to students and educators, requires a license. Alternative commercial CAD software: Solidworks
2. Image processing and analysis can be carried out with the users’ preferred analysis software. Here, all image analysis and processing were carried out using MATLAB 2021b (MathWorks), typically available on institution licenses. Open-source alternatives: Python, Fiji (ImageJ)
3. Stage, microscope, and camera were controlled by National Instrument Systems (NIS) Elements. Open-source alternatives: Micromanager versions 1.4 and 2.0
4. Single pressure controller, controlled by default software (Elveflow). Alternative: Fluigent’s microfluidic flow controller

All data and code examples used for the calibration and validation of this protocol are publicly available [19,29].

## Procedure

Below, we describe the step-by-step procedure for the fabrication, experimental setup, and calibration of the MCD. This device has been used to screen the chemotactic response of motile swimming bacteria to a logarithmically spaced concentration of a range of potential chemoattractants [19,21]. For the two-layer device, standard photolithography procedures [30,31] are used to fabricate two master molds. Subsequently, soft lithography with polydimethylsiloxane (PDMS) is used to assemble the MCD on a standard double-wide glass slide. The photomask designs are included in the Supplementary Material, and further data and example scripts are publicly available (see Software and Datasets). Photomasks were printed on mylar at a resolution of 25,400 dpi (Arnet Pro, formally CAD/Art Services Inc.).

**Outline of the protocol:**

- Fabricating microchannel molds via photolithography
- Fabrication of the master mold for the multi-height serial dilution layer
- Fabrication of the master mold for the single-height cell injection layer
- Silanization of the two master molds by vapor deposition
- PDMS soft lithography of the two master molds
- Assembly of the MCD
- Experimental procedure
- Calibration of flow rates

- **Fabricating microchannel molds via photolithography**
  1. Silicon wafer cleaning
    - Submerge and soak wafer in a separate glass Petri dish containing 50 mL of acetone for 3 min.
    - Submerge and soak wafer in a separate glass Petri dish containing 50 mL of isopropyl alcohol for 2 min.
    - Submerge and soak wafer in a separate glass Petri dish containing 100 mL of deionized water (DI H_2_O) for 2 min.
    - Remove wafer from DI H_2_O bath and rinse with a spray bottle containing DI H_2_O.
    - Dry wafer with compressed nitrogen.
    - Place clean wafer on a hotplate set to 150 °C for at least 15 min.
*Note: Before continuing to the photolithography step below (step A2), make sure the wafer has cooled to room temperature. Failing to do so may negatively impact photoresist film thickness during the spin coating step.*
  2. General photolithography protocol
    - Spin-coat the silicon wafer with SU-8 photoresist:
      i. Place the wafer on the spin coater using the wafer alignment tool to ensure the wafer is placed on the center of the chuck.
      ii. Pour SU-8 photoresist onto the center of the wafer, covering approximately a 50 mm diameter area according to manufacturer's recommendations.
*Note: Sufficient photoresist should be poured onto the wafer to ensure the photoresist completely coats the wafer after spin coating steps are completed below.*
      iii. Program and run spin coater with the following steps to form the desired photoresist film thickness:
      1) Spin wafer with an acceleration of 100 rpm/s up to 500 rpm and hold for 10 s to evenly spread the photoresist onto the wafer.
      2) Spin wafer with an acceleration of 300 rpm/s up to the necessary rotation speed (*S_i,i_*) to achieve the desired film thickness and hold for 30 s.
      3) Spin with a deceleration of 100 rpm/s to ramp down to 500 rpm, hold for 5 s, and complete the spin coating protocol.
    - Soft bake (also known as prebake):
      i. Place the wafer on a level preheated hotplate for 1 min at 65 °C.
      ii. Increase the hotplate temperature to 95 °C to ramp up the temperature of the wafer and hold for the duration specified in the manufacturer’s data sheets, which is based on the thickness of the photoresist.
      iii. Reduce the hotplate temperature back to 65 °C and remove the wafer after the temperature is achieved.
*Note: Steps A2b.ii and A2b.iii are optional but are performed to limit thermal stresses generated by changes in temperature.*
    - Load the wafer into the mask aligner, carefully aligning the photomask with the center of the wafer.
    - Transfer photomask pattern to SU-8 through UV exposure using mask aligner.
*Note: The time duration (t) of the UV exposure is set by the power output (P) of the mask aligner, and the required exposure energy (E) is set by the thickness of the photoresist based on the manufacturer’s protocol: t = E/P.*
    - Post-exposure bake (PEB)
      i. Place the wafer on a level preheated hotplate for 1 min at 65 °C.
      ii. Increase the hotplate temperature to 95 °C to ramp up the temperature of the wafer and hold for the duration specified by the manufacturer’s data sheets, which is based on the thickness of the photoresist.
      iii. Slowly reduce the hotplate temperature back to room temperature.
*Note: Steps A2e.i and A2e.iii are highly recommended to avoid thermal stresses generated by changes in temperature.*
    - Photoresist development
      i. Place the wafer inside a glass Petri dish and rinse with the photoresist developer (propylene glycol methyl ether acetate). Submerge the wafer in the developer and agitate the wafer frequently with wafer tweezers and/or laboratory rocker for at least 5–10 min, until features are fully developed.
      ii. Rinse the wafer first with isopropyl alcohol and then DI water until all traces of undeveloped photoresist are removed and the rinsing agents run clear. Dry gently with compressed air.
    - **Optional:** Hard bake. The hard bake step is recommended to increase the lifespan of the molds and/or to anneal cracks if any are observed when viewing the molds under a microscope.
      i. Place the wafer on a level hotplate set to 65 °C for 1 min.
      ii. Increase the hotplate to 100 °C and hold for 1 min.
      iii. Increase the hotplate to 150 °C and hold for 3 min.
*Note: Duration of step A2g.iii can vary if cracks are still visible*.
- **Fabricating the master mold for the multi-height dilution layer**
*Note: See “General notes: Microfabrication tolerances” for previous ranges of microfluidic tolerances.*
  1. Clean a silicon wafer based on step A1.
  2. Mount the wafer to the spin coater, apply the photoresist SU-8 3050, and program the spin coater described in step A2a where *S_1,1_* = 2,000 rpm for a target height of 90 µm.
  3. Soft bake the wafer with photoresist described in step A2b, baking the wafer at 95 °C for 45 min.
  4. Allow the wafer to cool gradually, then load wafer into the mask aligner with the first mask for layer 1 (main channel pattern).
  5. Transfer the pattern by exposing it for the appropriate duration (see note on step A2d).
  6. Move the wafer back to the hot plate and carry out the post-exposure bake (step A2e) at 95 °C for 5 min.
  7. Return the wafer to the spin coater, apply the photoresist SU-8 2025 on top of the baked features, and program the spin coater described in step A2a where *S_1,2_* = 4,200 rpm for a target height of 37 µm.
  8. Soft bake the wafer with photoresist described in step A2b, baking the wafer at 95 °C for 6 min.
  9. Allow the wafer to cool gradually, then load it into the mask aligner with the second mask containing the herringbone ridges for the dilution layer.
  10. Align the herringbone photomask with wafer by adjusting the wafer (x-y translation and rotation) until the alignment markers are placed correctly (Figure 1).
  11. Transfer the pattern by exposing for the appropriate duration (see note on step A2d).
  12. Move the wafer back to the hot plate and carry out the post-exposure bake (step A2e) at 95 °C for 12 min.
  13. Develop the wafer to remove the unexposed photoresist as described in step A2f and then hard bake as described in step A2g.
    **Figure 1.**
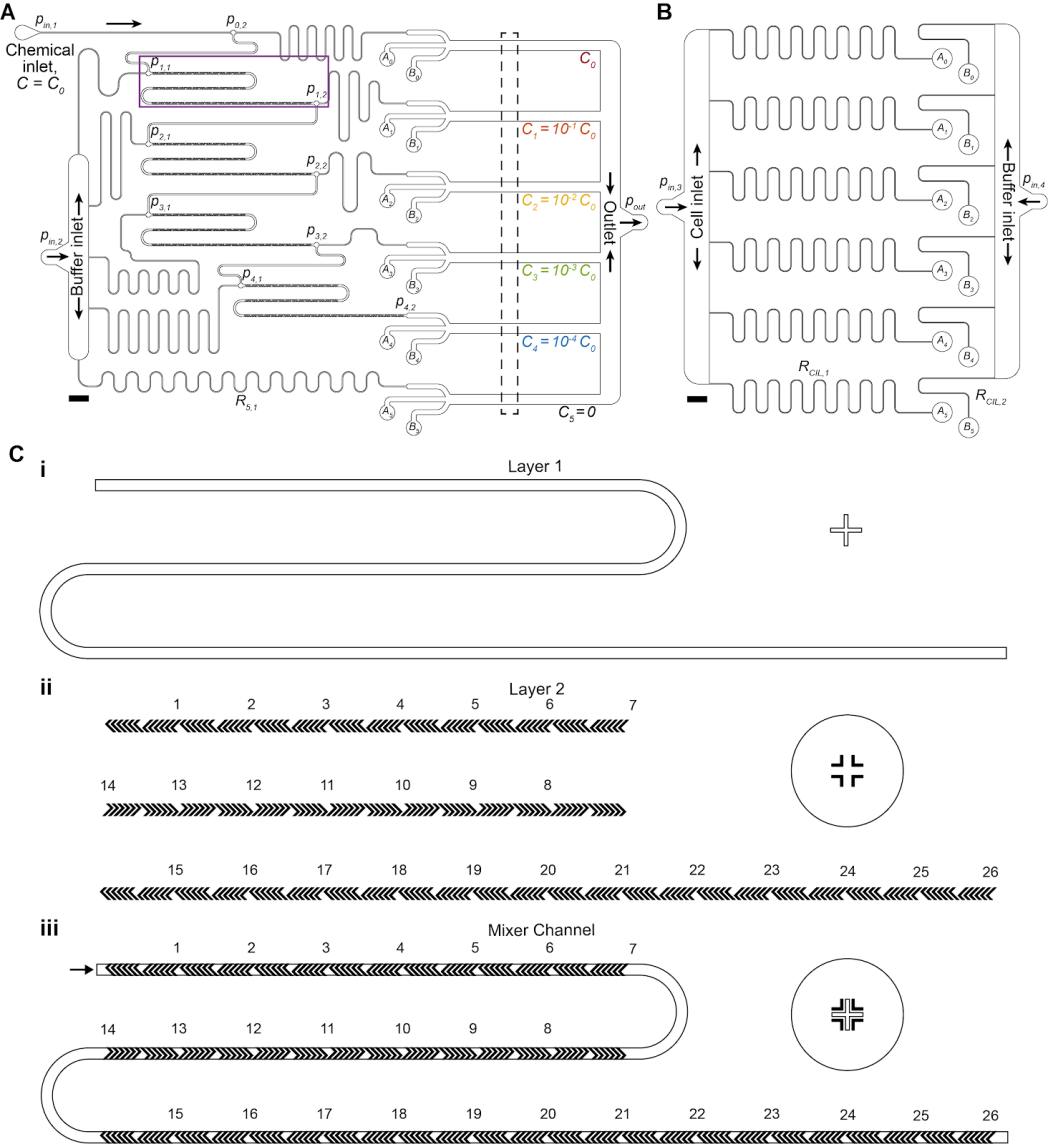
Multiplexed chemotaxis device (MCD) channel layout and multilayer photolithography using alignment markers. (A, B) Dilution layer (A) and cell injection layer (B) microchannel geometry scaled drawing. Scale bars 2 mm. (C) Multilayer photolithography was necessary to fabricate the herringbone ridges of the micromixer channels of the dilution layer. Alignment markers designed into the photomasks ensured proper orientation of the design. An inset of the microchannel design (magenta box from A) shows the alignment marker on the silicon wafer (i) used to align the herringbone ridge pattern within the photomask (ii) to ensure the ridges are placed in the correct location on the main channel (iii).
- **Fabricating the master mold of the single-height cell injection layer**
  1. Clean a silicon wafer based on step A1.
  2. Mount the wafer to the spin coater, apply the photoresist SU-8 3050, and program the spin coater described in step A2a where S2 = 2,550 rpm for a target height of 75 µm.
  3. Soft bake the wafer with photoresist described in step A2b, baking the wafer at 95 °C for 45 min.
  4. Allow the wafer to cool gradually, then load the wafer into the mask aligner with the photomask containing the cell injection layer pattern (Figure 1).
  5. Transfer the pattern by exposing for the appropriate duration (see note on step A2d).
  6. Move the wafer back to the hot plate and carry out the post-exposure bake (step A2e) at 95 °C for 8 min.
  7. Develop the wafer to remove the unexposed photoresist as described in step A2f, then hard bake as described in step A2g.
- **Silanization of the molds by vapor deposition**
**Critical:** Silanization of the wafers, especially the dilution layer master mold, will ensure easy release of the cast PDMS at the end of the soft lithography process. This step will need to be repeated periodically after several castings to maintain easy release, which is signaled by the casting becoming increasingly difficult to remove from the wafer. Use of this silanization agent does not result in any negative effects on microbes and has been used successfully with other biological microfluidic assays [32,33].
  1. Carefully remove the wafers from any containers, rinse first with isopropyl alcohol and then with DI H_2_O, and blow dry with nitrogen/compressed air.
  2. Load 1–2 drops of silanization agent (tridecafluoro-1,1,2,2-tetrahydrooctyl trichlorosilane) onto a glass slide, a small glass container, or a disposable weighing boat.
  3. Place the two wafers and silanization agent inside a vacuum desiccator for a minimum of 1 h.
*Note: The wafers can be silanized separately if required due to space constraints.*
**Caution:** Filters are recommended to prevent the silanization agent from entering the vacuum system/pump. Where possible, use a dedicated vacuum chamber for chemicals to prevent cross-contamination for other applications.
  4. Remove the wafers from the vacuum desiccator and place in fresh plastic Petri dishes for storage prior to soft lithography. Secure the edges of the wafers to the base of the Petri dishes with lab tape.
- **PDMS soft lithography of the two wafers**
  1. Prepare polydimethylsiloxane (PDMS) in a disposable container, e.g., plastic cup, in a 10:1 ratio (base:curing agent, see Recipe 1).
    - If the wafers have been previously cast, then approximately 20–30 g of PDMS base per wafer is required. Otherwise, if this is the first casting post-silanization, 80–100 g of PDMS base is recommended per wafer to ensure sufficient coverage of the entire Petri dish and wafer.
*Note: Excess PDMS will be pourable for approximately 12 h (depending on the exact curing agent ratio), so multiple casts can be completed with a sufficient quantity of prepared PDMS.*
    - Thoroughly mix the PDMS with a disposable implement (e.g., plastic spreader, plastic knife) for at least 1 min until the solution is opaque with bubbles.
*Note: A figure-of-eight motion is recommended for improved incorporation of the two components, with regular scraping of the sides and bottom of the container.*
  2. Place the mixed PDMS inside a vacuum desiccator and degas the mixture for at least 10 min until all bubbles are removed.
  3. Remove the PDMS from the vacuum chamber and gently pour over the two molds, tilting to ensure complete PDMS coverage of each wafer.
  4. Replace the wafers back inside the vacuum chamber and degas for approximately 10 min until most bubbles have been eliminated.
  5. Remove the wafers from the vacuum chamber.
**Optional:** Gently burst any remaining bubbles on the surface with gentle bursts of compressed air.
  6. Place the poured mold inside in a level oven at 60–70 °C for at least 1 h until the PDMS is fully cured.
*Note: The PDMS should be firm to the touch and not sticky when fully cured.*
  7. Carefully remove the PDMS slabs from the wafers by cutting around the edge of each layer with a sharp, precision knife until the PDMS perimeter has been fully cut. Carefully lift the edges of the slab away from the mold, working around the slab until it is fully free of the mold.
**Caution:** Care must be taken not to tear any of the PDMS features, particularly around the serpentine herringbone ridges of layer 1. The PDMS should release with relative ease; if not, repeat the silanization process (see section D).
  8. Cover both sides of each PDMS slab with Scotch tape to protect from dust.
  9. Carefully punch the tubing ports with a 1.5 mm biopsy punch (or equivalent tool) for both the dilution layer (chemical inlet, buffer inlet, *A_i_*, and *B_i_*) and cell injection later (cell inlet and buffer inlet) as shown in Figure 1A, B.
**Caution:** Punch the ports as perpendicular to the plane of the device as possible; sloped ports will make alignment of the two layers (see step F10) more difficult.
  10. Store the completed PDMS layers in a Petri dish prior to assembly.
**Pause point:** The procedure can be paused here indefinitely, until the devices are required for assembly.
- **Assembly of the MCD**
  1. Rinse the dilution layer PDMS with ethanol and then deionized water, blow drying with nitrogen/compressed air.
  2. Clean the PDMS with Scotch tape by repeatedly applying then removing tape from all surfaces of the PDMS, taking care to clean within individual features. Leave a layer of tape over the clean features to protect microchannels from dust before continuing to the next steps. Scotch tape is recommended as it does not leave a residue on the surfaces and remains easy to remove after a long time.
  3. Clean the glass slide (standard double-wide glass slide, 75 mm × 50 mm × 1 mm) with isopropyl alcohol scrubbing firmly with tissue (e.g., Kimtech wipes). Rinse with DI water, then blow-dry with nitrogen/compressed air. If necessary, the glass slide can be soaked in harsher treatments, e.g., hydrogen peroxide or standard hydrochloric acid cleaning processes to remove any residue and/or coatings from the surface.
  4. Place a double-wide glass slide and dilution layer PDMS feature-side up (removing any protective tape) inside a plasma oven and expose the surfaces to the plasma for 60 s.
  5. Remove glass and PDMS from the plasma oven using tweezers, taking care not to touch the exposed faces of either material. Invert the PDMS, so microchannels are facing down, and gently bring into contact with the glass slide (Figure 1A).
*Note: If air gaps get trapped between the glass slide and PDMS, lightly press the top of the PDMS with tweezers to remove.*
  6. Immediately after contact, place the partially assembled device on a hot plate at 110 °C for at least 30 min to promote covalent bonding.
**Inspection:** Under a microscope, inspect the microchannels to ensure no debris is blocking the microchannels. If debris is observed, attempt to remove it by flowing DI water through the channels; however, if the debris remains, it is recommended to cast a new PDMS layer instead.
**Pause point:** The protocol can be paused here indefinitely. It is advised and recommended that the first bonded layer is kept in a dust-free location (e.g., in a Petri dish) with Scotch tape covering the top of the PDMS to prevent dust accumulation in the inlets and on the bonding surface.
  7. Clean both PDMS surfaces: feature-side of cell injection PDMS and exposed top of the bonded dilution PDMS. Use Scotch tape to clean by repeatedly applying then removing tape from all surfaces of the PDMS, taking care to clean within individual features. Leave a layer of tape over the clean features for dust protection.
  8. Place glass-bonded dilution layer PDMS and cell injection layer PDMS feature-side up (removing any protective tape) inside a plasma oven and run for 60 s.
  9. Remove the partially assembled device and PDMS from the plasma oven using tweezers, taking care not to touch the exposed faces of either material.
  10. Invert the cell injection layer so the microchannels are facing downward. Carefully, by eye, align the features of cell injection layer with the punched ports of the dilution layer (Figure 1B) and then bring the two layers into contact.
**Critical:** At this stage, the PDMS-PDMS bond is partially reversible, so there is a margin (~3–5 s) for removing the cell injection layer if the two PDMS pieces are incorrectly aligned (Figure 1C, D). However, disturbing the bond could compromise the device’s integrity. It is recommended that steps F9–11 are repeated if the two layers are separated during alignment.
*Note: A microscope (e.g., stereomicroscope) can also be used to aid in alignment if necessary.*
  11. Place the glass-side down on a hot plate at 110 °C for at least 1 h to improve bond quality. Then, place the device on a 70 °C hotplate overnight. Store in dust-free condition.
- **Experimental procedure**
**Critical:** Careful loading and preparation of fluid connections is critical for successful operation of the MCD. It is essential, as with standard microfluidic techniques, that any air bubbles are removed from the device prior to experiments.
  1. Prepare 100 mL of 0.5% (w/v) BSA in DI H_2_O (see Recipe 2), vortex thoroughly, and then filter through a 0.2 μm syringe filter. Store in cool dark conditions until required.
  2. **Optional:** Place the fully assembled MCD inside a chamber under vacuum for 15 min to remove air from inside the device and PDMS.
*Note: The total time to degas the MCD in the vacuum desiccator depends on the overall thickness of the device. If unsure, an extended degassing period of 30 min is recommended.*
  3. Prepare four 20 mL syringes for gravity-fed filling of the MCD. For each syringe:
    - Remove the plunger and attach a 23G Luer lock needle.
    - Thread the end of 60 cm of Tygon tubing (henceforth referred to as tubing) onto the needle.
    - Attach each syringe (facing downward) into a lab flask grip stand at least 50 cm high.
  4. Immediately after removing the MCD from the vacuum chamber, quickly fill each syringe with the prepared BSA solution; once fluid begins to exit the tubing, connect the tubing to each inlet of the MCD (Figure 1A, Figure 2A).
    **Figure 2.**
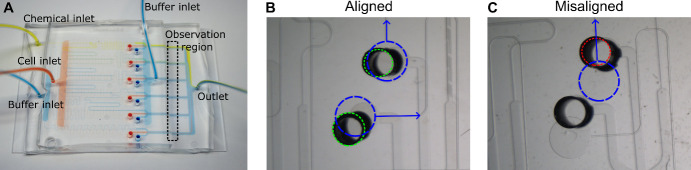
Multiplexed chemotaxis device assembly. (A) Shown with food coloring, successful assembly results in passive serial dilution and injection of the chemical solution with buffer from the dilution layer as well as the injection of buffer and cells from the cell injection layer. Dashed box corresponds to the observation region where experimental data is recorded. Scale bar, 5 mm. After assembly, inspection of the connection ports between the cell injection layer and dilution layer is necessary to ensure the MCD operates correctly. (B) Good alignment between the dilution layer (green dashed circle) and cell injection layer (blue dashed circle) is achieved when the punched holes in the dilution layer do not overlap with the labeled microchannels (blue arrows). (C) An example of misalignment in the intersection of the microchannel (blue arrow) of the cell injection layer (dashed blue circle) and the punched port of the dilution layer (red dashed circle), which will change the hydraulic resistance of the microchannel thus negatively altering the symmetry of stratified chemical, cell, and buffer solutions in the observation region (see Figure 3). Scale bar (B and C), 1 mm.
  5. Connect outlet waste tubing to the MCD and then clamp the tubing closed near the open end of the tubing once no more air bubbles can be seen exiting the device.
*Note: The hydrostatic pressure from the BSA syringes will slowly remove any air pockets/bubbles from the device. The BSA will pre-treat the surfaces to reduce adhesion of bacteria to the device.*
  6. Leave the device under gravity-pressure until no bubbles are visible within the device; allow at least 30–60 min or until the bubbles have disappeared. The required duration depends on the pressure applied and initial wetting of the microchannels. If a desiccator is not used to remove air from the device and PDMS (see optional step G2), the device can be placed under gravity-pressure overnight to slowly remove any bubbles. Proceed with step G7 whilst step G6 completes.
**Critical:** Bubbles stuck to the channel walls will compromise the device operation, and thus enough time should be allowed for the air bubbles to be absorbed in the PDMS prior to experiments.
*Notes:*
    - *More pressure can be applied by either (i) increasing the height of the syringes above the device, or (ii) reinserting the plungers and using an elastic band to exert pressure on each of the syringes.*
    - *Alternative methods for degassing the MCD are described in the General Notes section.*
  7. Prepare experimental solutions for the four inlets (Figure 1) connected to the pressure controller.
*Note: See previous applications for examples for preparation of specific experimental solutions [19,21].*
    - Dilution layer—chemical inlet: solution of chemostimulus dissolved into buffer solution (e.g., artificial seawater, minimal motility media), concentration *C_0_.*
    - Dilution layer—buffer inlet: buffer solution.
    - Cell injection layer—cell inlet: cells washed and resuspended into buffer solution.
    - Cell injection layer—buffer inlet: buffer solution.
  8. Disconnect the MCD from all BSA syringes, making sure to leave a droplet on top of each inlet/outlet to facilitate attachment of the inlet tubing.
  9. Attach each inlet tubing with the device in turn:
    - Briefly turn on the pressure controller to form a small droplet on the end of the tubing, then stop the flow.
**Critical:** Bring the tubing into contact with the droplet on top of the corresponding inlet of the MCD, maintaining a fluid-fluid connection at all times to prevent air being pushed into the device. If the end of the tubing exits the droplet before connection is established, reset the droplet using the pressure controller.
    - Push the tubing into the inlet, using tweezers if necessary to ensure a firm hold. Test that the tubing is inserted correctly with a light tug on the tubing; the tubing should remain firmly attached to the MCD.
  10. Insert outlet tubing (40 cm) into the outlet and connect it to a waste container.
  11. Secure the MCD to the microscope stage.
  12. Ensure the liquid–air interfaces of each fluid inlet container and outlet container are level (i.e., at the same relative heights). Raise/lower individual Falcon tubes as necessary to achieve this.
*Note: The liquid–air interface of each container should be level across all containers, so they have the same absolute pressure resulting in a more accurate applied pressure from the pressure controller. This process eliminates the potential for residual hydrostatic-driven flows.*
  13. Setup microscope acquisition. Locate a mid-plane observation point in each experimental channel (Figure 1A). An experimental time of approximately 10 min is recommended to start. Slower diffusing chemoattractants might require longer acquisition times due to a longer persisting gradient. The magnification should be selected to allow for imaging of the full channel width in a single field of view, which is dependent on the available scientific camera (typically expect a magnification between 4× and 10×).
  14. Start the pressure controller using pressure ratios from calibration (see section H). The designed applied pressure ratio between the dilution layer (*p_in,1-2_*) and cell injection layer (*p_in,3-4_*) are 2:1, though minor pressure adjustments will be necessary to have symmetric stratified chemical, cell, and buffer solutions in the observation region (see Figure 3) due to minor variations in the replica mold manufacturing processes [19].
    **Figure 3.**
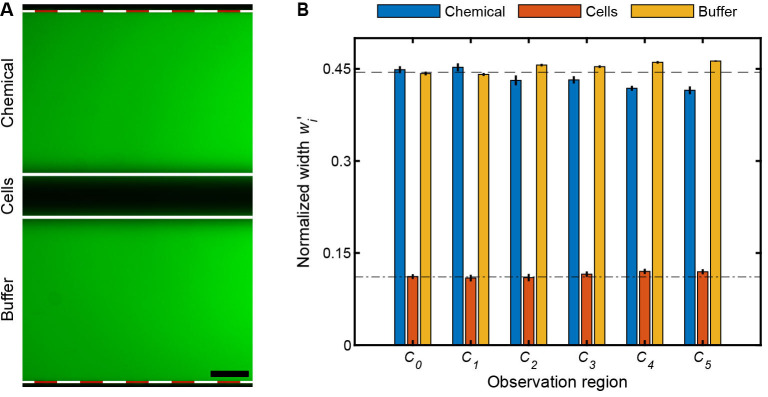
Multiplexed chemotaxis device (MCD) validation and calibration. (A) Shown with fluorescein (green) and DI H_2_O, the MCD is functioning properly when the stratified chemical, cell, and buffer solutions are symmetric in the observation region. Scale bar, 0.1 mm. (B) Normalized measured widths, $w_{i}^{'}=w_{i}/W$ , of the chemical, cell, and buffer streams illustrate their pronounced symmetry in each observation channels (*C_0-5_*, Figure 1A); where *w_i_* is the width of fluid stream (A; solid white lines) and *W* the total width of the intensity profile (A; dashed white-red lines). The bar plots and error bars indicate the mean and standard deviation of wi' for three separate driving pressures of the dilution layer and cell injection layer: (i) 100 mbar and 70 mbar, (ii) 150 mbar and 105 mbar, and (iii) 200 mbar and 140 mbar for *p_in,1-2_* and *p_in,3-4_*, respectively. To achieve symmetric laminar flow, the applied pressure, *p_in,3-4_*, was tuned. The slight deviations from the designed inlet pressures are due to a variety of experimental conditions such as (i) variations in the fabricated channel height of the dilution layer and cell injection layer master molds and/or (ii) variations in the inlet tubing lengths that supply the solutions to the MCD. These deviations are fixed per set of master molds; hence, flow rate calibration only needs to be carried out once per set of master molds.
  15. After at least 2 min of continuous flow, simultaneously stop the pressure controller and clamp close to the open end of the tubing connected waste outlet. Then, begin acquisition.
  16. After acquisition is completed, unclamp the waste outlet and cut the crimped tubing. Then, restart the flow and repeat steps G14–15 for as many technical replicates as required. Biological replicates can be easily conducted by replacing the cell suspension and restarting the flow.
- **Calibration of flow rates**
**Critical:** Calibration of the MCD only needs to be done with one complete device per set of wafers. Subsequent devices from the same wafers will perform identically, provided they are assembled correctly. The purpose of this calibration step is to establish the correct pressure ratio between the two layers to account for minor deviations in geometry due to the microfabrication processes. The designed pressures are *p_in,1-2_* = 100 mbar, *p_in,3-4_* = 50 mbar.
  1. Prepare 100 mL of 0.1 mM fluorescein solutions in DI H_2_O and 100 mL of DI water (see Recipes 3 and 4).
  2. Setup the MCD as per section G, flowing the 0.1 mM fluorescein solution through both the chemical and buffer inlet of the dilution layer, DI H_2_O through the cell inlet of the cell injection layer, and 0.1 mM fluorescein through the buffer inlet of the cell injection layer.
  3. Prepare the microscope for fluorescence microscopy with a GFP filter cube (for excitation of the fluorescein). Select a magnification that allows for imaging of the full width of the observation channel (1 mm) in a single field of view, typically in the range 4–10×.
  4. Turn on the pressure controller at the designed pressure ratios (*p_in,1-2_* = 100 mbar, *p_in,3-4_* = 50 mbar; Figure 4).
*Note: The observation regions will now have three distinct laminar fluid layers visible: two bright fluorescent bands from the chemical inlet of the dilution layer and the buffer inlet of the cell injection layer width (w_1,3_). The center width (w_2_) contains DI H_2_O from the cell inlet.*
  5. Acquire an image in each observation region (Figure 3).
  6. Adjust the pressure of the cell injection layer (*p_in,3-4_*, Figure 4) until *w_1_ ≈ w_3_* and then acquire a second image at each observation region (Figure 3).
    Precise widths for each fluid stream can be easily measured using the spatial intensity across the width of the observation region channel, either in the microscope software or in post-processing.
**Critical:** These pressure ratios will be robust across all devices made from a particular set of molds as long as the viscosity of the solutions is the same, and the absolute pressures can be increased as long as the relative pressure ratio (*p_in,1-2_/p_in,3-4_*, Figure 4) remains constant. See previous applications [19] for examples of the device operating at different absolute pressures with a constant pressure ratio.
    **Figure 4.**
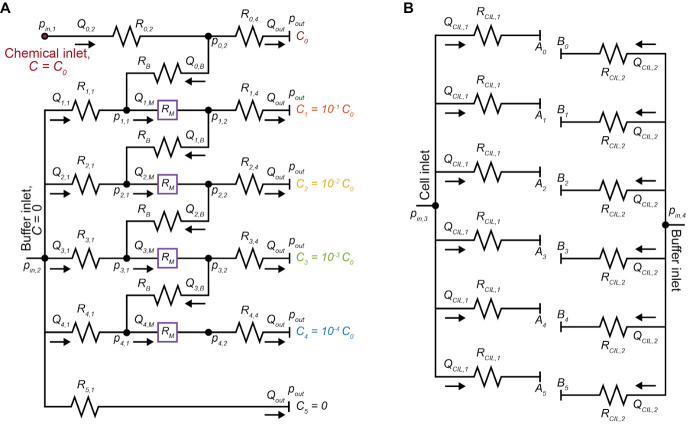
Multiplexed chemotaxis device (MCD) design. Circuit representation of the dilution layer (A) and cell injection layer (B) of the MCD. The pressure at each node (*p_i_*), microchannel volumetric flow rate (*Q_i_*), and microchannel hydraulic resistance (*R_i_*) must be known before designing and fabricating the MCD.

## Data analysis

A full description of data analysis methods has been previously described [19,21] and data analysis from previous applications and example calibration data are publicly available [29]. Alternatively, image analysis can be easily conducted with the software package of choice (e.g., Python, MATLAB, ImageJ) with commonly available image processing toolboxes. In summary, images were processed by removing the average (mean) background and applying a spatial bandpass filter; then, features were identified to sub-pixel accuracy. With the cell locations identified, the chemotactic behavior can be visualized through a variety of methods. Previous examples include heatmaps of the conditional probability [19,21] *P(y|t)*, accumulation toward the boundaries [19,21], and migration coefficients calculated from average cell distance from the center of the channel [15,34,35].

## Validation of protocol

This protocol has been used and validated in the following research articles:

- Stehnach et al. [19]. Multiplexed microfluidic screening of bacterial chemotaxis. *eLife*.
  - Fabrication and calibration: Figure 1, Figure 1 Figure Supplements 1–4
  - Validation: Figure 2, Figure 2 Figure Supplement 1
  - Applications: Figure 3 and Figure 4
  - Dataset [29]
- Henshaw et al. [21]. Early stage viral infection of cyanobacteria drives marine bacterial chemotaxis. *bioRxiv*.
  - Applications: Figure 4

## General notes and troubleshooting

**General notes**

1. Alternative technique to assist in the PDMS-PDMS bonding.
  After treating the two PDMS microchannels in the plasma oven (step F8), a small drop of DI H_2_O can be placed on the top of the dilution layer PDMS before aligning the cell injection PDMS [36]. This allows for the two PDMS pieces to slide into place to ensure they are properly aligned.
2. Microfabrication tolerances.
  The microfabrication process can vary due to a range of factors including age and temperature of the SU-8 and accuracy of the spin coater. Therefore, there will be deviations in the final channel heights away from the designed target heights. It is crucial that the height of the microchannel remains constant across the wafer; a systematic deviation away from the target height is more desirable than spatially varying mold heights across the master mold. In previous applications of this protocol [19,21], the target and achieved channel heights were:
  - Dilution layer main channel: Target, 90 μm; Achieved, 90–94.5 μm.
  - Herringbone ridges of the dilution layer: Target, 37 μm; Achieved, 37–38.5 μm.
  - Cell injection layer: Target, 75 μm; Achieved, 71–73 μm.
    The MCD will perform robustly despite these variations if each master mold is calibrated once prior to use (as outlined in the protocol).
3. Hydraulic circuit and microchannel design.
  Designing the MCD using hydraulic circuits (Figure 4): The Hagen-Poiseuille law describes the flow characteristics for an incompressible fluid in a channel with laminar flow as:
*Δp = QR_H_*
  where *Δp* is the pressure drop along the channel, *Q* is the volumetric flow rate, and *R_H_* is the hydraulic resistance defined by a combination of the geometrical properties of the channel and mechanical properties of the fluid [37]. The channel lengths within the provided hydraulic circuits for this protocol (Supplementary Materials) are determined by setting the following: channel height, applied inlet pressures *p_in,1-2_*, outlet pressure *p_out_*, outlet flow rate *Q_out_*, resistance of the bridge channel *R_B_*, resistance of the outlet *R_4,4_*, and resistance of the herringbone micromixer channel *R_M_*. A set of simultaneous equations can then be established and solved for the unknown flow rates, pressures, and hydraulic resistances (and corresponding channel dimensions). This process will need to be repeated if the user wishes to alter features of the MCD such as dilution ratios, linear versus logarithmic dilution, or to increase/decrease the number of observation channels for example. For a complete description of this process, which is out of the scope of this protocol, please see the original work [19].
4. Standard microfluidic procedures.
  This protocol builds upon standard microfluidic techniques, which will be familiar to experienced users. However, for less experienced users, we recommend reviewing the cited literature throughout this protocol and additionally the following materials [38–40]:
  - O’Laughlin et al. [38]. Fabrication of Microfluidic Devices for Continuously Monitoring Yeast Aging. *Bio-protocol*.
  - Taly et al. [39]. *Microfluidics Diagnostics: Methods and Protocols.* Springer
  - Bruggeman et al. [40]. Microfluidics and fluorescence microscopy protocol to study the response of C. elegans to chemosensory stimuli. *STAR Protocols*
5. Alternative methods for degassing/filling of microfluidic devices.
  Users can use any preferred method for removing air bubbles from the microchannels, such as the described “gravity feed” method in the main text. Below, we outline two commonly used alternative strategies to achieve the desired degassing and subsequent filling of the microfluidic device:
  - Flowing DI H_2_O through the device at a high flow rate with some perturbations to dislodge any bubbles stuck to the channel geometry, using either a syringe or peristaltic pump.
  - Fully submerge the device in DI H_2_O, then place in a vacuum desiccator. The vacuum will draw the air out from the device and pull the liquid into the microchannels.

## Supplementary information

The following supporting information can be downloaded here:

1. SI_Material_MCD_CAD_Designs_AllLayers.dwg: CAD Art files containing the photomasks designs outlined in this protocol.
